# 1209. Let’s take a time-out: Implementing a standardized process to assess the clinical use of vancomycin for pharmacists

**DOI:** 10.1093/ofid/ofad500.1049

**Published:** 2023-11-27

**Authors:** Jennifer Oderinde, Anthony Chiang, Alicia Juska, O L U W A D A M I L O L A A ADEYEMI

**Affiliations:** Swedish Hospital NorthShore University HealthSystem, Chicago, Illinois; Advocate Lutheran General Hospital, Evanston, IL; Swedish Hospital NorthShore University HealthSystem, Chicago, Illinois; Swedish Hospital Part of NorthShore University HealthSystem, Chicago, Illinois

## Abstract

**Background:**

Vancomycin is often started empirically for its reliability with gram-positive pathogens, particularly *Methicillin Resistant Staph aureus* (MRSA). Consequently, it is often over-prescribed. Antimicrobial stewardship programs (ASP) have used time-outs to ensure therapies are tailored to the patient with proper indication and coverage. This study aims to evaluate the impact of a standardized vancomycin time-out to optimize utilization and safety.

**Methods:**

This is a pre-/post-implementation study conducted at a community teaching hospital. The study was separated into a pre-implementation phase (July-September 2022), implementation/teaching phase (October 2022), and post-implementation phase (November 2022-February 2023). Study subjects included inpatient adults on vancomycin for longer than 48 hours. Patients who are immunocompromised, hospice, or on a vancomycin regimen prior to admission were excluded.

Primary outcomes include (1) percent appropriate vancomycin continued after time-out, (2) percent inappropriate vancomycin discontinued during time-out, and (3) time to de-escalation. Additional outcomes include vancomycin days of therapy (DOT) per 1000 patient days, hospital length of stay, and acute kidney injury (AKI) incidence rate.

This study looked at 182 patients in the retrospective arm and 214 patients in the prospective arm. Preliminary data was initially presented at the Illinois Pharmacy Residency Conference when only 195 total patients had been reviewed.

**Results:**

There was a statistically significant difference seen in percent appropriate vancomycin continued after time-out, with 57% and 40.2% seen in the prospective and retrospective arm respectively. The percent inappropriate vancomycin discontinued during time-out was also statistically significant with 69.9% and 38.8% respectively. With a time-out, average days to discontinuation for continued inappropriate regimens, and overall vancomycin DOT were decreased. There was no significant difference seen in safety outcomes including AKI incidence rates.

**Conclusion:**

Pharmacy driven time-outs are useful for promoting antimicrobial stewardship and can contribute to decreased vancomycin use,

**Disclosures:**

**All Authors**: No reported disclosures

